# Assessing Priorities in a Statewide Cardiovascular and Diabetes Health Collaborative Based on the Results of a Needs Assessment: Cross-Sectional Survey Study

**DOI:** 10.2196/55285

**Published:** 2024-04-12

**Authors:** Elizabeth A Beverly, Sarah Koopman-Gonzalez, Jackson Wright, Kathleen Dungan, Harini Pallerla, Rose Gubitosi-Klug, Kristin Baughman, Michael W Konstan, Shari D Bolen

**Affiliations:** 1 Department of Primary Care Ohio University Heritage College of Osteopathic Medicine Athens, OH United States; 2 Department of Medicine Case Western Reserve University Cleveland, OH United States; 3 Department of Medicine Ohio State University College of Medicine Columbus, OH United States; 4 Department of Family and Community Medicine University of Cincinnati College of Medicine Cincinnati, OH United States; 5 Department of Family and Community Medicine Northeast Ohio Medical University Rootstown, OH United States; 6 Center for Health Care Research and Policy MetroHealth Medical Center Cleveland, OH United States

**Keywords:** health collaborative, cardiovascular disease, type 2 diabetes, needs assessment

## Abstract

**Background:**

The Ohio Cardiovascular and Diabetes Health Collaborative (Cardi-OH) unites general and subspecialty medical staff at the 7 medical schools in Ohio with community and public health partnerships to improve cardiovascular and diabetes health outcomes and eliminate disparities in Ohio’s Medicaid population. Although statewide collaboratives exist to address health improvements, few deploy needs assessments to inform their work.

**Objective:**

Cardi-OH conducts an annual needs assessment to identify high-priority clinical topics, screening practices, policy changes for home monitoring devices and referrals, and preferences for the dissemination and implementation of evidence-based best practices. The results of the statewide needs assessment could also be used by others interested in disseminating best practices to primary care teams.

**Methods:**

A cross-sectional survey was distributed electronically via REDCap (Research Electronic Data Capture; Vanderbilt University) to both Cardi-OH grant-funded and non–grant-funded members (ie, people who have engaged with Cardi-OH but are not funded by the grant).

**Results:**

In total, 88% (103/117) of Cardi-OH grant-funded members and 8.14% (98/1204) of non–grant-funded members completed the needs assessment survey. Of these, 51.5% (53/103) of Cardi-OH grant-funded members and 47% (46/98) of non–grant-funded members provided direct clinical care. The top cardiovascular medicine and diabetes clinical topics for Cardi-OH grant-funded members (clinical and nonclinical) were lifestyle prescriptions (50/103, 48.5%), atypical diabetes (38/103, 36.9%), COVID-19 and cardiovascular disease (CVD; 38/103, 36.9%), and mental health and CVD (38/103, 36.9%). For non–grant-funded members, the top topics were lifestyle prescriptions (53/98, 54%), mental health and CVD (39/98, 40%), alcohol and CVD (27/98, 28%), and cardiovascular complications (27/98, 28%). Regarding social determinants of health, Cardi-OH grant-funded members prioritized 3 topics: weight bias and stigma (44/103, 42.7%), family-focused interventions (40/103, 38.8%), and adverse childhood events (37/103, 35.9%). Non–grant-funded members’ choices were family-focused interventions (51/98, 52%), implicit bias (43/98, 44%), and adverse childhood events (39/98, 40%). Assessment of other risk factors for CVD and diabetes across grant- and non–grant-funded members revealed screening for social determinants of health in approximately 50% of patients in each practice, whereas some frequency of depression and substance abuse screening occurred in 80% to 90% of the patients. Access to best practice home monitoring devices was challenging, with 30% (16/53) and 41% (19/46) of clinical grant-funded and non–grant-funded members reporting challenges in obtaining home blood pressure monitoring devices and 68% (36/53) and 43% (20/46) reporting challenges with continuous glucose monitors.

**Conclusions:**

Cardi-OH grant- and non–grant-funded members shared the following high-priority topics: lifestyle prescriptions, CVD and mental health, family-focused interventions, alcohol and CVD, and adverse childhood experiences. Identifying high-priority educational topics and preferred delivery modalities for evidence-based materials is essential for ensuring that the dissemination of resources is practical and useful for providers.

## Introduction

### Background

Cardiovascular disease (CVD) is the leading cause of death in the United States [1] and worldwide, representing 32% of all global deaths or 17.9 million deaths annually [2]. In 2021, the age-adjusted CVD mortality rate was 204.7 per 100,000 Ohio residents, which is 17.8% higher than the national average [3]. Numerous risk factors contribute to Ohio’s higher rates of CVD, including tobacco use, hypertension, and diabetes. For example, in 2023, Ohio had the fourth highest smoking rate in the United States, with 21% of the population (2.4 million people) smoking [4]. Among adults with CVD in Ohio, 73% also have hypertension, and 69% have hyperlipidemia [5]. Similarly, data from 2016 show that adults with CVD in Ohio are more likely to have diabetes, cancer, kidney disease, asthma, and chronic obstructive pulmonary disease compared to those without CVD [5]. For these reasons, statewide approaches that address modifiable cardiometabolic risk factors are needed to facilitate the dissemination and implementation of evidence-based best practices for health care teams.

In 2017, the Ohio Department of Medicaid (ODM) funded the development of the Ohio Cardiovascular and Diabetes Health Collaborative (Cardi-OH) [6]. Cardi-OH is a statewide health collaborative that unites the 7 medical schools in Ohio to improve cardiovascular and diabetes health outcomes and reduce disparities in Ohio’s Medicaid population. Health collaboratives mobilize community and public health partnerships to identify and solve health problems [7]. Specifically, Cardi-OH brings together primary care physicians, specialists, pharmacists, physician assistants, nurse practitioners, nurses, dietitians, and social scientists affiliated with the 7 medical schools as well as representatives from the ODM and Ohio’s Medicaid Managed Care plans to share their expertise to accelerate learning and the implementation of best practices. The ODM also separately funds statewide quality improvement projects focused on paired implementation in which Cardi-OH resources may be used [8,9].

### Objectives

To prioritize the dissemination of topics each year, Cardi-OH conducts an annual needs assessment to identify educational foci for CVD risk reduction and type 2 diabetes management. Previous literature on needs assessments by regional and statewide health collaboratives has focused broadly on factors that influence health in the community or on a review of cardiovascular health or diabetes statistics for a particular region [10,11]. Our needs assessment differs by eliciting primary care team members’ most important considerations for implementing evidence-based best practices for both CVD risk reduction and type 2 diabetes. We present findings from the 2021 to 2022 needs assessment survey that identifies the prioritized topics, use of selected screening practices, and selected barriers to the dissemination and implementation of evidence-based best practices, which were used to develop the Cardi-OH materials for the 2022-2023 academic year. These findings may provide guidance for health care systems, professional organizations, payers, community-based organizations, and other health collaboratives looking to prioritize activities for CVD risk reduction and type 2 diabetes management.

## Methods

### Ethical Considerations

Ethics approval for the needs assessment was obtained from the Case Western Reserve University Institutional Review Board (study STUDY20180486). The research was classified as exempt and, therefore, did not require signed informed consent. In accordance with the Belmont principle of respect for persons, all Cardi-OH members were given the opportunity to choose whether to participate in the assessment. Furthermore, in compliance with federal, state, and local laws and regulations for human participants, we ensured that our research met the requirements set forth in the regulations on public welfare in Part 46 of Title 45 of the Code of Federal Regulations, the principles set forth in the Belmont Report, and the Helsinki Declaration of 1975. All study data were deidentified before analysis. Respondents received no human participant compensation for taking part in the needs assessment.

### Research Design

We conducted a descriptive cross-sectional needs assessment with all members of the Cardi-OH community. Specifically, we administered a confidential electronic survey to identify important clinical topics, screening practices, policy changes for home monitoring devices and referrals, and preferences for the dissemination modality of evidence-based best practice materials. We elected to conduct a needs assessment because it is a systematic approach used to identify the priorities of a group and determine its capacity to address the needs of the population being served.

### Participants

During the 2021-2022 academic year, Cardi-OH included a total of 1321 members. Of these 1321 members, 117 (8.86%) were grant-funded members affiliated with 1 of the 7 Ohio medical schools. For this noncompetitive statewide grant, grant-funded members included direct care providers and public health professionals specializing in CVD and type 2 diabetes. These grant-funded members were responsible for identifying, producing, and disseminating the latest evidence-based cardiovascular and diabetes best practices. The remaining 91.14% (1204/1321) of members were non–grant-funded members. Non–grant-funded members included community providers or stakeholders who engaged with Cardi-OH by registering through Cardi-OH–sponsored events (ie, statewide webinars and Cardi-OH Extension for Community Healthcare Outcomes [ECHO] clinics), registering through the Cardi-OH website, or engaging with Cardi-OH via alignment efforts with other statewide partners such as regional professional associations and community-based organizations. There were no exclusion criteria for joining Cardi-OH as a non–grant-funded member. Both grant-funded and non–grant-funded Cardi-OH members were tracked in a REDCap (Research Electronic Data Capture; Vanderbilt University) [12,13] database to record membership, event attendance, and program evaluation data.

### Measures

Cardi-OH consists of 5 large teams: the executive team (ie, principal investigators from the 7 medical schools), team best practices (ie, the team that reviews and synthesizes evidence and national guidelines for dissemination), team ECHO (ie, the team that develops curricular content for 12-week web-based case-based learning series), the communications team (ie, the team responsible for branding, disseminating materials, and maintaining the website), and the data and evaluation team (ie, the team responsible for evaluating the success and effectiveness of the events and materials produced by Cardi-OH). The data and evaluation team comprises 12 experts in quantitative and qualitative methodologies representing 5 of the 7 medical schools. The goal of the data and evaluation team is to establish a set of metrics designed to measure the effectiveness of Cardi-OH. Every year, the data and evaluation team solicits input from all teams for CVD- and type 2 diabetes–related topics for an annual needs assessment. We have conducted needs assessments in previous years; however, this was the first year we administered the needs assessment to both grant-funded and non–grant-funded Cardi-OH members. Demographic questions are carried over from one year to the next, but topical questions are new with each needs assessment. These questions include lists of suggested topics that Cardi-OH members rank as high-priority educational topics to develop events and materials for in the upcoming year. The 2021 to 2022 needs assessment survey consisted of 24 questions; clinical members had to answer all 24 (100%) questions whereas nonclinical members had to answer only 5 of the 24 (21%) questions. For nonclinical members, the needs assessment took approximately 3 to 5 minutes to complete; for clinical members, the needs assessment took approximately 10 to 15 minutes to complete. Clinical members answered more questions because the survey included questions about the direct care provided, screening practices, comfort level with topics, and perceived difficulty ordering remote monitoring devices. While the focus of the needs assessment is clinical, we also include nonclinical members’ priorities to promote alignment with public health professionals across the state. Effective collaboration between clinical and public health professionals is essential to improve cardiovascular and type 2 diabetes health outcomes.

To establish face and content validity, the data and evaluation team reviewed and rated each question to determine whether it was necessary, useful, and relevant to be included in the needs assessment. The needs assessment was then piloted on February 24, 2022, with 2 primary care physicians to identify any weak or irrelevant questions; no questions were removed after the pilot test. Reliability and validity testing was not conducted because no specific constructs (eg, knowledge, attitudes, and beliefs) were measured using this assessment.

### Data Collection

The 2022 Cardi-OH needs assessment was disseminated electronically via REDCap to all Cardi-OH members. REDCap is a secure electronic data capture program designed for collecting survey data [12,13]; the program was hosted by Case Western Reserve University. The survey opened on March 29, 2022, and closed on April 22, 2022. Participation in this study was voluntary. All Cardi-OH members were sent 2 email reminders.

### Statistical Analysis

Descriptive statistics were used to describe the survey participants. The frequencies of individual question responses were calculated by clinical and nonclinical status as well as by grant- and non–grant-funded status. For the purposes of the analysis, clinical providers were defined as any Cardi-OH member who provided direct clinical care to patients. Statistical significance was defined as *P*<.05. All analyses were conducted using SPSS statistical software (version 28.0; IBM Corp).

## Results

### Cardi-OH Survey Participants

A total of 88% (103/117) of grant-funded members completed the needs assessment (Table 1). Of these, 51.5% (53/103) provided direct clinical care, of whom 79% (42/53) identified as physicians. More than half (30/53, 57%) of clinical providers practiced in a primary care setting, and 43% (23/53) worked in internal medicine. Providers estimated that 41.5% (SD 19.8%) of patients were enrolled in Medicaid.

**Table 1:** Ohio Cardiovascular and Diabetes Health Collaborative (Cardi-OH) grant- and non–grant-funded members’ demographic characteristics.

Question			Grant-funded members (n=103)		Non–grant-funded members (n=98)		Chi-square (*df*) or Fisher exact test		*P* value
**All Cardi-OH members: in what sector are you employed? (Select all that apply), n (%)**									
	Academic	88 (85.4)		27 (27.6)		68.7 (1)		<.001	
	Health care	48 (46.6)		56 (57.1)		2.2 (1)		.14	
	Health plan or insurer	1 (1)		6 (6.1)		—^a^		.06	
	Pharmaceutical or manufacturing	1 (1)		1 (1)		—		>.99	
	Nonprofit	9 (8.7)		15 (15.3)		—		.19	
	Philanthropic	0 (0)		0 (0)		—		—	
	Private	0 (0)		3 (3.1)		—		.11	
	Public	18 (17.5)		20 (20.4)		—		.60	
	Research or policy or not academic	10 (9.7)		2 (2)		0.2 (1)		.03	
	Other	0 (0)		2 (2)		—		.24	
**All Cardi-OH members: do you provide direct clinical care to patients as part of a clinical team? This could be serving as a medical assistant, social worker, physician, etc, n (%)**							0.1 (1)		.67
	Yes	53 (51.5)		46 (46.9)					
	No	48 (46.6)		52 (53.1)					
**Clinical Cardi-OH members: what is your clinical role?, n (%)^b^**							17.8 (10)		.06
	Physician	42 (79.2)		24 (52.2)					
	Nurse practitioner	1 (1.9)		5 (10.9)					
	Physician assistant	2 (3.8)		1 (2.2)					
	RN^c^	1 (1.9)		6 (13)					
	LPN^d^	0 (0)		1 (2.2)					
	Medical assistant	0 (0)		2 (4.3)					
	Registered dietitian	1 (1.9)		1 (2.2)					
	Social worker	1 (1.9)		3 (6.5)					
	Psychologist	0 (0)		1 (2.2)					
	Pharmacist	4 (7.5)		1 (2.2)					
	Other	0 (0)		1 (2.2)					
**Clinical Cardi-OH members: in what setting do you practice?, n (%)^b^**							3.9 (2)		.14
	Primary care	30 (56.6)		33 (71.7)					
	Specialty care	20 (37.7)		9 (19.6)					
	Other	3 (5.7)		4 (8.7)					
**Clinical Cardi-OH members: in what specialty area do you work?, n (%)^b^**							3.3 (5)		.64
	Family medicine	15 (28.3)		17 (37)					
	Internal medicine	23 (43.4)		14 (30.4)					
	Pediatrics	3 (5.7)		5 (10.9)					
	Geriatrics	1 (1.9)		1 (2.2)					
	Other	10 (18.9)		9 (19.6)					
**Clinical Cardi-OH members: how would you describe the geographic setting where you primarily practice? (Select all that apply), n (%)^b^**									
	Urban	42 (79.2)		30 (65.2)		2.4 (1)		.12	
	Suburban	18 (34)		16 (34.8)		0.0 (1)		.93	
	Rural	6 (11.3)		8 (17.4)		0.7 (1)		.39	
	Other	1 (1.9)		0 (0)		0.8 (1)		.35	
Clinical Cardi-OH members: approximately what percentage of the patients you serve are enrolled in Medicaid? (%), mean (SD)			41.5 (19.8)		51.7 (29.3)		–1.9 (87)		.06
**Clinical Cardi-OH members: how often do you provide clinical care?, n (%)^b^**							7.5 (3)		.06
	>75% of the time	9 (17)		19 (41.3)					
	50%-75% of the time	16 (30.2)		8 (17.4)					
	25%-49% of the time	11 (20.8)		8 (17.4)					
	<25% of the time	17 (32.1)		11 (23.9)					

^a^Fisher exact test.

^b^Grant-funded members: n=53; non–grant-funded members: n=46.

^c^RN: registered nurse.

^d^LPN: licensed practical nurse.

A total of 8.14% (98/1204) of non–grant-funded members completed the needs assessment. Of these, 47% (46/98) reported providing direct clinical care, of whom 52% (24/46) identified as physicians. More than two-thirds (33/46, 72%) of the non–grant-funded providers practiced in a primary care setting, and 37% (17/46) worked in family medicine. Providers estimated that 51.7% (SD 29.3%) of their patients were enrolled in Medicaid.

### Top-Rated Cardiovascular Medicine Topics

Cardi-OH members were asked to choose their top topics of interest for cardiovascular medicine (Multimedia Appendix 1). For all grant-funded members (n=103; clinical and nonclinical), the top cardiovascular medicine topics were lifestyle prescriptions (50/103, 48.5%; Multimedia Appendix 1), atypical diabetes (38/103, 36.9%), COVID-19 and CVD (38/103, 36.9%), mental health and CVD (38/103, 36.9%), and alcohol and CVD (32/103, 31.1%). Among clinical grant-funded members, the highest-rated topics were atypical diabetes (29/53, 55%), calcium scoring (26/53, 49%), and special cases of CVD and diabetes (24/53, 45%).

For all non–grant-funded members (n=98), the top cardiovascular medicine topics were lifestyle prescriptions (53/98, 54%), mental health and CVD (39/98, 40%), alcohol (27/98, 28%), and cardiovascular complications (27/98, 28%). The top 3 topics among clinical non–grant-funded members were lifestyle prescriptions (27/46, 59%), mental health and CVD (22/46, 48%), and alcohol and CVD (20/46, 43%).

### Top-Rated Topics Related to Social Determinants of Health

Regarding social determinants of health (SDoHs), grant-funded members (clinical and nonclinical) selected their top topics: (1) weight bias and stigma (44/103, 42.7%; Multimedia Appendix 2), (2) family-focused interventions for CVD and diabetes (40/103, 38.8%), (3) adverse childhood experiences and their association with CVD and diabetes (37/103, 35.9%), and (4) implicit bias and CVD (37/103, 35.9%). For clinical grant-funded members, the highest-rated topics were family-focused interventions for CVD and diabetes (24/53, 45%), weight bias and stigma (22/53, 42%), and peer support interventions for CVD and diabetes (19/53, 36%).

Non–grant-funded members selected (1) family-focused interventions for CVD and diabetes (51/98, 52%), (2) implicit bias and CVD (43/98, 44%), and (3) adverse childhood experiences and their association with CVD and diabetes (39/98, 40%). Among clinical non–grant-funded members, the highest-rated topics were adverse childhood experiences and their association with CVD and diabetes (19/46, 41%), gender disparities in CVD (17/46, 37%), and implicit bias and CVD (17/46, 37%).

### Screening Practices for SDoHs

More than half (28/53, 53%) of clinical grant-funded members felt “very” or “extremely” comfortable screening for SDoHs (Table 2). Almost half (21/53, 40%) screened for SDoHs when their patients appeared to have barriers, and 25% (13/53) screened at every visit. Only 23% (12/53) of clinical grant-funded members were “very” or “extremely” confident that they could address their patients’ SDoHs. The most common methods for screening for SDoHs were verbal (30/53, 57%), web (21/53, 40%), paper (8/53, 15%), and others (2/53, 4%).

**Table 2:** Ohio Cardiovascular and Diabetes Health Collaborative grant- and non–grant-funded members’ responses to the questions on screening.

Questions				Clinical grant-funded members (n=53), n (%)		Clinical non–grant-funded members (n=46), n (%)		Chi-square (*df*)		*P* value
**Questions on screening for SDoHs^a^**										
	**How comfortable do you feel screening for SDoHs in adults with type 2 diabetes?**							5.8 (5)		.33
		Extremely comfortable	13 (25)		12 (26)					
		Very comfortable	15 (28)		11 (24)					
		Moderately comfortable	18 (34)		11 (24)					
		Slightly comfortable	4 (8)		5 (11)					
		Not at all comfortable	2 (4)		7 (15)					
	**How often do you screen for SDoHs?**							8.2 (7)		.31
		Annually	6 (11)		12 (26)					
		Every visit	13 (25)		11 (24)					
		Only when the patient appears to have barriers	21 (40)		10 (22)					
		Never	1 (2)		1 (2)					
		N/A^b^	5 (9)		7 (15)					
		Other	5 (9)		2 (4)					
		Do not know	2 (4)		2 (4)					
	**How confident are you that you can address the SDoHs of your patients (eg, referrals)?**							9.9 (6)		.13
		Extremely confident	7 (13)		6 (13)					
		Very confident	5 (9)		13 (28)					
		Moderately confident	23 (43)		13 (28)					
		Slightly confident	12 (23)		9 (20)					
		Not at all confident	3 (6)		5 (11)					
		N/A	2 (4)		0 (0)					
	**What method do you use to screen for SDoHs? (Select all that apply)**									
		Paper	8 (15)		18 (39)		7.4 (1)		.007	
		Web	21 (40)		20 (43)		0.2 (1)		.70	
		Verbal	30 (57)		25 (54)		0.2 (1)		.82	
		Other	2 (4)		0 (0)		1.8 (1)		.18	
		Do not know	5 (9)		5 (11)		0.1 (1)		.81	
**Questions on screening for behavioral health**										
	**Do you routinely screen patients for depression?**							8.4 (4)		.08
		All patients are screened	36 (68)		35 (76)					
		Certain populations are screened	5 (9)		3 (7)					
		No	5 (9)		3 (7)					
		Do not know	7 (13)		0 (0)					
		N/A	0 (0)		5 (11)					
	**How often do you screen patients for depression?**							5.3 (4)		.26
		Annually	14 (26)		17 (37)					
		Every visit	20 (38)		19 (41)					
		Only when the patient appears depressed	4 (8)		0 (0)					
		Other	3 (6)		1 (2)					
		N/A	0 (0)		0 (0)					
		Do not know	0 (0)		0 (0)					
	**How often are you able to connect Medicaid patients with needed behavioral health services?**							5.5 (5)		.36
		Always	7 (13)		12 (26)					
		Often	16 (30)		15 (33)					
		Sometimes	19 (36)		12 (26)					
		Rarely	1 (2)		2 (4)					
		Never	0 (0)		0 (0)					
		N/A	8 (15)		5 (11)					
		Do not know	2 (4)		0 (0)					
**Questions on screening for substance abuse**										
	**How often do you screen patients for substance abuse?**							6.3 (6)		.39
		Annually	12 (23)		16 (35)					
		Every visit	12 (23)		13 (28)					
		Only when the patient shows signs of substance abuse	11 (21)		4 (9)					
		My practice does not screen for substance abuse	3 (6)		3 (7)					
		Other	5 (9)		1 (2)					
		N/A	7 (13)		7 (15)					
		Do not know	3 (6)		2 (4)					
	**How often are you able to connect Medicaid patients with needed treatment for substance abuse disorders?**							11.3 (6)		.08
		Always	5 (9)		12 (26)					
		Often	7 (13)		9 (20)					
		Sometimes	17 (32)		14 (30)					
		Rarely	4 (8)		4 (9)					
		Never	0 (0)		0 (0)					
		N/A	14 (26)		7 (15)					
		Do not know	5 (9)		0 (0)					

^a^SDoH: social determinant of health.

^b^N/A: not applicable.

Among the clinical non–grant-funded members, 50% (23/46) felt “very” or “extremely” comfortable screening for SDoHs. A total of 22% (10/46) screened for SDoHs when their patients appeared to have barriers, and 24% (11/46) screened at every visit. A total of 41% (19/46) of clinical non–grant-funded members were “very” or “extremely” confident that they could address their patients’ SDoHs. The most common methods for screening for SDoHs were verbal (25/46, 54%), web (20/46, 43%), and paper (18/46, 39%).

The only significant difference between clinical grant- and non–grant-funded members pertained to the screening methods for SDoHs. Specifically, clinical non–grant-funded members used paper more frequently than did grant-funded members (*χ*^2^_1_=7.4; *P*=.007). No other differences were observed in screening practices for SDoHs.

### Screening Practices for Depression

For depression screening, 68% (36/53) of clinical grant-funded members indicated that all patients were screened, 9% (5/53) screened only certain populations, 13% (7/53) selected *not applicable*, and 9% (5/53) did not screen (Table 2). Regarding the frequency of depression screening, 38% (20/53) screened at every visit, 26% (14/53) screened annually, 8% (4/53) screened when the patient appeared depressed, and 6% (3/53) selected *other*. Only 13% (7/53) of clinical grant-funded members specified that they were always able to connect their Medicaid patients with needed behavioral health services, 30% (16/53) reported that they were able to do so often, 36% (19/53) reported that they were able to do so sometimes, 2% (1/53) reported that they were able to do so rarely, 4% (2/53) did not know, and 15% (8/53) selected *not applicable*.

Among clinical non–grant-funded members, 76% (35/46) indicated that all patients were screened for depression, 7% (3/46) screened only certain populations, and 11% (5/46) selected *not applicable*. Regarding the frequency of depression screening, 41% (19/46) screened at every visit, 37% (17/46) screened annually, and 2% (1/46) selected *other*. Approximately one-quarter (12/46, 26%) of clinical non–grant-funded members specified that they were always able to connect their Medicaid patients with needed behavioral health services, 33% (15/46) reported that they were able to do so often, 26% (12/46) reported that they were able to do so sometimes, 4% (2/46) reported that they were able to do so rarely, and 11% (5/46) selected *not applicable*.

No differences were observed in behavioral health screening practices between clinical and nonclinical grant-funded members (Table 2).

### Screening Practices for Substance Abuse

For substance abuse screening, 23% (12/53) of clinical grant-funded members screened at every visit, 23% (12/53) screened annually, 21% (11/53) screened when patients showed signs of substance abuse, 6% (3/53) did not know, 6% (3/53) did not screen, 13% (7/53) selected *not applicable*, and 9% (5/53) selected *other* (Table 2). Regarding their ability to connect Medicaid patients with needed treatment for substance abuse disorders, 9% (5/53) reported being able to do so always, 13% (7/53) reported being able to do so often, 32% (17/53) reported being able to do so sometimes, 8% (4/53) reported being able to do so rarely, 9% (5/53) did not know, and 26% (14/53) selected *not applicable*.

Among clinical non–grant-funded members, 28% (13/46) screened for substance abuse at every visit, 35% (16/46) screened annually, 9% (4/46) screened when patients showed signs of substance abuse, 4% (2/46) did not know, 7% (3/46) did not screen, 15% (7/46) selected *not applicable*, and 2% (1/46) selected *other*. Regarding their ability to connect Medicaid patients with needed treatment for substance abuse disorders, 26% (12/46) indicated that they were able to do so always, 20% (9/46) reported that they were able to do so often, 30% (14/46) reported that they were able to do so sometimes, 9% (4/46) reported that they were able to do so rarely, and 15% (7/46) selected *not applicable*.

No differences were observed in substance abuse screening practices between clinical and nonclinical grant-funded members (Table 2).

### Perceived Difficulty Obtaining Home Monitoring Devices and Making Referrals

Clinical Cardi-OH members were asked about their perceived difficulty obtaining specific home monitoring devices and making referrals because these were areas that Medicaid was specifically trying to address. Of the 53 clinical grant-funded members, 16 (30%; Table 3) perceived obtaining home blood pressure monitors as “moderately,” “very,” or “extremely” difficult, followed by 7 (13%) who perceived obtaining glucometers as “moderately” or “very” difficult and 36 (68%) who perceived obtaining continuous glucose monitoring (CGM) devices as “moderately,” “very,” or “extremely” difficult. Finally, 34% (18/53) of clinical grant-funded members found referring a Medicaid patient to diabetes self-management education and support (DSMES) “moderately,” “very,” or “extremely” difficult.

**Table 3:** Ohio Cardiovascular and Diabetes Health Collaborative grant- and non–grant-funded members’ responses to questions on obtaining home monitoring devices and making referrals.

Questions		Clinical grant-funded members (n=53), n (%)	Clinical non–grant-funded members (n=46), n (%)	Chi-square (*df*)		*P* value	
**How difficult is it for you to obtain home BP^a^ monitors for your Medicaid patients with hypertension?**					4.0 (5)		.55
	Extremely difficult	2 (4)	2 (4)				
	Very difficult	4 (8)	5 (11)				
	Moderately difficult	10 (19)	12 (26)				
	Slightly difficult	13 (25)	8 (17)				
	Not at all difficult	7 (13)	10 (22)				
	N/A^b^	17 (32)	9 (20)				
**How difficult is it for you to obtain glucometers for your Medicaid patients with type 2 diabetes?**					1.8 (4)		.77
	Extremely difficult	0 (0)	0 (0)				
	Very difficult	2 (4)	1 (2)				
	Moderately difficult	5 (9)	3 (7)				
	Slightly difficult	18 (34)	12 (26)				
	Not at all difficult	17 (32)	20 (43)				
	N/A	11 (21)	10 (22)				
**How difficult is it for you to obtain CGM^c^ devices for your Medicaid patients with type 2 diabetes?**					16.4 (6)		.01
	Extremely difficult	6 (11)	3 (7)				
	Very difficult	9 (17)	11 (24)				
	Moderately difficult	21 (40)	6 (13)				
	Slightly difficult	2 (4)	9 (20)				
	Not at all difficult	1 (2)	4 (9)				
	N/A	13 (25)	13 (28)				
**How difficult is it for you to refer a Medicaid patient to DSMES^d^ ?**					3.5 (6)		.75
	Extremely difficult	2 (4)	2 (4)				
	Very difficult	3 (6)	2 (4)				
	Moderately difficult	13 (25)	7 (15)				
	Slightly difficult	9 (17)	6 (13)				
	Not at all difficult	14 (26)	17 (37)				
	N/A	12 (23)	11 (24)				

^a^BP: blood pressure.

^b^N/A: not applicable.

^c^CGM: continuous glucose monitoring.

^d^DSEMS: diabetes self-management education and support.

Of the 46 clinical non–grant-funded members, 19 (41%; Table 3) found obtaining home blood pressure monitors “moderately,” “very,” or “extremely” difficult, followed by 4 (9%) who perceived obtaining glucometers as “moderately” or “very” difficult and 20 (43%) who perceived obtaining CGM devices as “moderately,” “very,” or “extremely” difficult. Finally, 24% (11/46) of clinical non–grant-funded members perceived referrals to DSMES as “moderately,” “very,” or “extremely” difficult.

Clinical grant-funded members differed from clinical non–grant-funded members in perceived difficulty ordering CGM devices (*χ*^2^_6_=16.4; *P*=.01; Table 3). No other differences were observed in perceived difficulty obtaining home monitoring devices and referring to DSMES.

### Preferences for Dissemination of Evidence-Based Materials

Finally, both grant- and non–grant-funded members were asked about their preferences for the delivery modality of evidence-based best practice materials. The Cardi-OH best practices team produces a variety of materials, including a monthly newsletter, capsules (1-page summaries of best practices ready for implementation in clinical care), currents (half-page summaries of recent peer-reviewed articles describing the latest advances in medicine), best practice documents (web-based tools and resources), Cardi-OH ECHO clinic didactic recordings, and podcasts. All Cardi-OH members were asked to select their top 3 delivery modalities; thus, the percentages exceed 100% (Table 4).

**Table 4:** Ohio Cardiovascular and Diabetes Health Collaborative (Cardi-OH) grant- and non–grant-funded members’ preferences for the delivery modality of evidence-based best practice materials.

Question: from the list of Cardi-OH materials, SELECT UP TO THREE that are of most interest to you	Grant-funded members (n=103), n (%)	Non–grant-funded members (n=98), n (%)	Chi-square (*df*)	*P* value
Newsletters (monthly updates highlighting new best practice content and other timely information about the collaborative, including links to web documents, capsules, and currents)	72 (69.9)	57 (58.2)	3 (1)	.08
Capsules (brief 1-page summaries of best practices that are ready to be implemented in clinical care)	68 (66)	47 (48)	6.6 (1)	.01
Currents (brief half-page summaries of recent articles describing the latest advances in medicine or clinical practice related to cardiovascular health)	49 (47.6)	42 (42.9)	0.4 (1)	.50
Best practice documents (web-based tools and resources to help clinicians aid patients in managing cardiovascular health)	49 (47.6)	65 (66.3)	7.2 (1)	.007
Cardi-OH ECHO^a^ clinic didactic recordings (brief videos of presentations to improve content knowledge and share evidence-based best practices)	17 (16.5)	24 (24.5)	1.9 (1)	.16
Podcasts (audio recordings highlighting national, state, and local leaders discussing timely topics for primary care clinicians)	35 (34)	24 (24.5)	2.1 (1)	.14

^a^ECHO: Extension for Community Healthcare Outcomes.

Grant-funded members preferred these materials in the following order: newsletters (72/103, 69.9%), capsules (68/103, 66%), currents (49/103, 47.6%), best practice documents (49/103, 47.6%), podcasts (35/103, 34%), and ECHO didactic recordings (17/103, 16.5%). Non–grant-funded member preferences were as follows: best practice documents (65/98, 66%), newsletters (57/98, 58%), capsules (47/98, 48%), currents (42/98, 43%), podcasts (24/98, 24%), and ECHO didactic recordings (24/98, 24%).

Grant-funded members preferred capsules more than non–grant-funded members (68/103, 66% vs 47/98, 48%; *χ*^2^_1_=6.7; *P*=.01), and non–grant-funded members preferred best practice documents more than grant-funded members (65/98, 66% vs 49/103, 47.6%; *χ*^2^_1_=6.7; *P*=.01). No other differences were observed in preferences for delivery modality.

### Best Practice Events and Materials

Events and materials were created based on the following top clinical topics: lifestyle prescriptions (website document: *Implementing Lifestyle Prescriptions in Primary Care*), CVD and mental health (website document: *Mental Health and Chronic Conditions: Treating the Whole Patient to Improve Self-Care*), and alcohol and CVD (podcast: *Addressing Unhealthy Alcohol Use: Strategies for Primary Care*). The top SDoH topics included weight bias and stigma (ECHO didactic recording: *Obesity: Bias and Discrimination*), adverse childhood experiences (capsule: *Adverse Childhood Experiences and Cardiovascular Disease Risk*), and family-focused interventions (capsule [*Tips to Improve Family Support for Heart-Healthy Living*] and website document [*Family Support as a Key Component of Cardiovascular Disease Prevention and Care*]). To address preferences for dissemination, these topics were presented in a variety of modalities. Additional events and materials were created to cover the following topics that were rated as being of moderate interest in the needs assessment: COVID-19 (statewide webinar), sleep disorders (statewide webinar and podcast), smoking cessation (capsule), atypical diabetes (capsule), supplements (website document and podcast), the role of clinical pharmacists (website document), lipids and statin use (podcast), complications (current), and disability (capsule and podcast). Importantly, all the events and materials are publicly available on the Cardi-OH website.

## Discussion

### Principal Findings

In this cross-sectional, descriptive needs assessment, we surveyed clinical and nonclinical grant- and non–grant-funded members of the Cardi-OH community. The purpose of the needs assessment was to identify high-priority topics for the dissemination of evidence-based best practices to Ohio’s health care providers. We also evaluated our clinical members’ screening practices regarding critical topic areas important to health equity and CVD care, perceived difficulties obtaining home monitoring devices and referrals to DSMES as Medicaid payers were actively working to address these barriers, and preferences for the dissemination of evidence-based best practice materials. Overall, both clinical grant- and non–grant-funded members prioritized the following CVD-related topics: lifestyle prescriptions, CVD and mental health, and alcohol and CVD. For SDoH-related topics, clinical grant- and non–grant-funded members prioritized family-focused interventions and adverse childhood experiences. Regarding screening for SDoHs, half of clinical grant- and non–grant-funded members felt “very” or “extremely” comfortable screening for SDoHs, and they used a variety of modalities to screen. In addition, 68% (36/53) of clinical grant-funded members and 76% (35/46) of clinical non–grant-funded members screened all patients for depression, with most screening at every visit. Regarding substance abuse screening, 23% (12/53) of clinical grant-funded members and 28% (13/46) of clinical non–grant-funded members screened at every visit. In all screening categories, connecting with resources to address these areas was challenging. When asked about difficulty with ordering home monitoring devices, clinical grant- and non–grant-funded members perceived ordering home blood pressure monitors (16/53, 30% vs 19/46, 41%, respectively) and CGMs (36/53, 68% vs 20/46, 43%, respectively) as “moderately,” “very,” or “extremely” difficult. Finally, Cardi-OH grant- and non–grant-funded members’ top 3 delivery modalities for evidence-based materials were newsletters, capsules, and best practice documents. Interestingly, grant-funded members were more likely to prefer capsules, and non–grant-funded members were more likely to prefer best practice documents.

### Comparison With Prior Work

Dissemination and implementation of evidence-based best practices for CVD and diabetes are critical to address the leading cause of morbidity and mortality worldwide. Best practices in CVD care include a wide range of factors such as preventive management [14,15]. Needs assessments help organizations identify problems, gaps in programming or content, and strategies to prioritize resources. Our needs assessment identified specific needs and gaps for each Cardi-OH team to inform their development of evidence-based best practice events and materials for the following year. For example, team best practices used the findings to create a website document on lifestyle prescriptions and a capsule on adverse childhood experiences. Because medicine is an information-based science and clinical care requires frequent information seeking, understanding the needs and preferences of providers is essential [16]. Preferences for dissemination were noted, and prioritized topics were covered in website documents and capsules, 2 of the most preferred delivery modalities, although we often try to use multiple modalities due to differences in learning styles and preferences. These preferences align with previous research examining health care providers’ information needs. Specifically, accessible web-based resources and summaries that synthesize evidence-based materials facilitate providers’ information-seeking behaviors [16]. Thus, asking what Cardi-OH members prefer regarding both topics and dissemination modalities may lead to increased engagement with the collaborative. Over time, we anticipate that increased engagement with the collaborative will increase the dissemination of evidence-based best practices to health care providers across the state. Increased dissemination of best practices improves the quality of care [17], standardizes care across providers and settings [18], reduces health expenditures [19], and increases efficiency in health care [20]. Best practices achieve these outcomes by reducing complications, decreasing hospitalizations, and preventing mortality [21,22].

Health collaboratives such as Cardi-OH are partnerships between health care providers, health care organizations, academic institutions, health plans and insurers, public health, government agencies, and other public and private stakeholders that work together to improve the Quadruple Aim: (1) improving the health of the population, (2) improving the patient experience, (3) reducing costs, and (4) improving care team well-being. Health collaboratives achieve this aim through collective learning, shared data, and diffusion of innovation [23,24]. Importantly, health collaboratives can take many forms and focus on different issues. For example, they may focus on a specific health condition, such as Cardi-OH’s focus on CVD and diabetes, or on care delivery or patient safety. Importantly, the success of a health collaborative hinges on members working together to leverage their collective expertise, resources, and authority to address complex challenges in health care. The key to leveraging expertise, resources, and authority is identifying the priorities of a collaborative and determining the group’s capacity to address the needs of the population being served. One way to identify a collaborative’s strengths, challenges, and priorities is through an annual needs assessment [25]. Importantly, annual needs assessments should be updated to capture context changes, needs, and priorities. Finally, needs assessments can build leadership and group cohesion and facilitate community involvement with health collaboratives [25].

The findings of our needs assessment can be used as a guide or template for other health collaboratives to identify high-priority educational topics in their region. We outline an approach that involves engaging with both clinical and nonclinical stakeholders to ensure that our collaborative’s priorities are aligned with our state’s needs. Similarly, our prioritized topics may be of interest to other organizations and primary care providers given the high prevalence of CVD and type 2 diabetes in the United States and worldwide. Furthermore, the events and materials we produce are publicly available to anyone at no cost. Sharing resources such as those created by Cardi-OH promotes additional partnerships to leverage the expertise and capabilities of multiple organizations.

### Limitations

Limitations include the sample size, limited use of inferential statistics, participant self-selection, self-reported data, and lack of patient perceptions. The response rate among Cardi-OH grant-funded members was high (103/117, 88%); however, among non–grant-funded members, it was only 8.14% (98/1204). The low response rate among non–grant-funded members introduced a bias between responders and nonresponders that we could not control for in the analysis. Future work should include efforts to increase the response rate of non–grant-funded members. Second, the self-reported findings may be susceptible to selection and social desirability biases. To minimize bias, the researchers informed participants that their responses were confidential. Furthermore, the researchers emphasized the voluntary nature of participation and explicitly informed the respondents that their responses had no bearing on their professional standing. Finally, the needs assessment did not assess patient perceptions. The current scope of Cardi-OH is to identify, produce, and disseminate the latest evidence-based cardiovascular and diabetes best practices to primary care providers in Ohio. Expansion of the needs assessment to include patient needs and preferences could be considered in future assessments by the collaborative to identify gaps, improve decision-making, understand treatment options, and improve health outcomes.

### Conclusions

Accelerating the learning of best practices in cardiovascular and diabetes care is essential to improve health outcomes and eliminate disparities. Over time, continuous learning of evidence-based best practices improves the quality of care [17], standardizes care across providers and settings [18], reduces health expenditures [19], and increases efficiency [20]. As medicine continues to develop new treatments and technologies, the importance of accelerating the learning of evidence-based best practices will increase, and health collaboratives such as Cardi-OH should play a critical role in keeping providers up-to-date on the latest developments in their field. Moreover, health collaboratives identify implementation gaps that arise between best practice research studies and frontline provision of care, allowing for communication and advocacy to address gaps in access to necessary resources (eg, home monitoring devices) and advanced care referrals. Our findings can be used to guide similar efforts by health care systems, professional organizations, payers, community-based organizations, and other health collaboratives looking to prioritize activities in CVD risk reduction and type 2 diabetes management.

## Appendix

Multimedia Appendix 1 Ohio Cardiovascular and Diabetes Health Collaborative members’ top-rated cardiovascular disease (CVD)–related topics by clinical and nonclinical grant- (n=103) and non–grant-funded (n=98) members.

Multimedia Appendix 2 Ohio Cardiovascular and Diabetes Health Collaborative members’ top-rated social determinant of health–related topics by clinical and nonclinical grant- (n=103) and non–grant-funded (n=98) members.

## Abbreviations

Cardi-OH: Ohio Cardiovascular and Diabetes Health Collaborative
CGM: continuous glucose monitoring
CVD: cardiovascular disease
DSMES: diabetes self-management education and support
ECHO: Extension for Community Healthcare Outcomes
ODM: Ohio Department of Medicaid
REDCap: Research Electronic Data Capture
SDoH: social determinant of health

## Data Availability

The data sets generated during and analyzed during this study are available from the corresponding author on reasonable request.
