# Supplementary material for: Assessing Priorities in a Statewide Cardiovascular and Diabetes Health Collaborative Based on the Results of a Needs Assessment: Cross-Sectional Survey Study
Source: JMIR Form Res. 2024 Apr 12;8:e55285. doi: 10.2196/55285 (PMC11053386; doi:10.2196/55285)
Supplement: Multimedia Appendix 1 [file formative_v8i1e55285_app1.docx]

Multimedia Appendix 1. Ohio Cardiovascular and Diabetes Health Collaborative members’ top-rated cardiovascular disease (CVD)–related topics by clinical and nonclinical grant- (n=103) and non–grant-funded (n=98) members.

| Topic | Granted-Funded Members (n=103), n (%) | Non-Grant-Funded Member (n=98), n (%) |
| --- | --- | --- |
| Alcohol and CVD | 32 (31.1) | 27 (27.6) |
| Atypical diabetes | 38 (36.9) | 26 (26.5) |
| Calcium scoring | 31 (30.1) | 21 (21.4) |
| Clinical pharmacists and CVD | 14 (13.6) | 16 (16.3) |
| CVD complications | 24 (23.3) | 27 (27.6) |
| COVID-19 and CVD | 38 (36.9) | 23 (23.5) |
| COVID-19 vaccines | 10 (9.7) | 8 (8.2) |
| Heart failure | 19 (18.4) | 26 (26.5) |
| Lifestyle prescriptions | 50 (48.5) | 53 (54.1) |
| Lipids | 12 (11.7) | 19 (19.4) |
| Mental health and CVD | 38 (36.9) | 39 (39.8) |
| Nontraditional risk factors of CVD | 18 (17.5) | 15 (15.3) |
| Older adults and CVD | 21 (20.4) | 12 (12.2) |
| Postpartum follow-up and CVD | 18 (17.5) | 19 (19.4) |
| Remote monitoring of CVD and diabetes | 10 (9.7) | 21 (21.4) |
| Sleep disorders and CVD | 25 (24.3) | 22 (22.4) |
| Smoking cessation | 14 (13.6) | 15 (15.3) |
| Special cases of CVD and diabetes | 29 (28.2) | 21 (21.4) |
| Supplements & CVD | 18 (17.5) | 13 (13.3) |
| Vaccine recommendations | 13 (12.6) | 10 (10.2) |
