# Supplementary material for: Assessing Priorities in a Statewide Cardiovascular and Diabetes Health Collaborative Based on the Results of a Needs Assessment: Cross-Sectional Survey Study
Source: JMIR Form Res. 2024 Apr 12;8:e55285. doi: 10.2196/55285 (PMC11053386; doi:10.2196/55285)
Supplement: Multimedia Appendix 2 [file formative_v8i1e55285_app2.docx]

Multimedia Appendix 2. Ohio Cardiovascular and Diabetes Health Collaborative members’ top-rated social determinant of health–related topics by clinical and nonclinical grant- (n=103) and non–grant-funded (n=98) members. ACE: adverse childhood experience; CVD: cardiovascular disease.

| Topic | Granted-Funded Members (n=103), n (%) | Non-Grant-Funded Member (n=98), n (%) |
| --- | --- | --- |
| Adverse Childhood Experiences (ACEs) & CVD | 37 (35.9) | 39 (39.8) |
| Disability & CVD | 29 (28.2) | 22 (22.4) |
| Family-Focused CVD Interventions | 40 (38.8) | 51 (52.0) |
| Gender Disparities in CVD | 34 (33.0) | 26 (26.5) |
| Implicit Bias & CVD | 37 (35.9) | 43 (43.9) |
| Incarceration, Community Transition, & CVD risk | 24 (23.3) | 22 (22.4) |
| Intimate Partner Violence & CVD | 9 (8.7) | 7 (7.1) |
| Peer Support Interventions & CVD | 27 (26.2) | 34 (34.7) |
| Weight Bias & Stigma | 44 (42.7) | 38 (38.8) |
